# Two's Company, Three's a Crowd: Experimental Evaluation of the Evolutionary Maintenance of Trioecy in *Mercurialis annua* (Euphorbiaceae)

**DOI:** 10.1371/journal.pone.0035597

**Published:** 2012-04-19

**Authors:** Laura E. Perry, John R. Pannell, Marcel E. Dorken

**Affiliations:** 1 Department of Biology, Trent University, Peterborough, Ontario, Canada; 2 Department of Plant Sciences, University of Oxford, Oxford, United Kingdom; 3 Department of Ecology and Evolution, University of Lausanne, Lausanne, Switzerland; Norwegian University of Science and Technology, Norway

## Abstract

Trioecy is an uncommon sexual system in which males, females, and hermaphrodites co-occur as three clearly different gender classes. The evolutionary stability of trioecy is unclear, but would depend on factors such as hermaphroditic sex allocation and rates of outcrossing vs. selfing. Here, trioecious populations of *Mercurialis annua* are described for the first time. We examined the frequencies of females, males and hermaphrodites across ten natural populations and evaluated the association between the frequency of females and plant densities. Previous studies have shown that selfing rates in this species are density-dependent and are reduced in the presence of males, which produce substantially more pollen than hermaphrodites. Accordingly, we examined the evolutionary stability of trioecy using an experiment in which we (a) indirectly manipulated selfing rates by altering plant densities and the frequency of males in a fully factorial manner across 20 experimental plots and (b) examined the effect of these manipulations on the frequency of the three sex phenotypes in the next generation of plants. In the parental generation, we measured the seed and pollen allocations of hermaphrodites and compared them with allocations by unisexual plants. In natural populations, females occurred at higher frequencies in denser patches, a finding consistent with our expectations. Under our experimental conditions, however, no combination of plant densities and male frequencies was associated with increased frequencies of females. Our results suggest that the factors that regulate female frequencies in trioecious populations of *M. annua* are independent of those regulating male frequencies (density), and that the stable co-existence of all three sex phenotypes within populations is unlikely.

## Introduction

The majority of flowering plants are hermaphroditic, but separate sexes (dioecy) have evolved numerous times [1]–[3]. How these transitions have occurred, and the extent to which hermaphrodite phenotypes can persist with males and/or females, have long puzzled evolutionary biologists [4]. The evolution of dioecy usually proceeds via the gynodioecy pathway, in which females become established in populations of hermaphrodites (yielding gynodioecy – the co-occurrence of females and hermaphrodites [3], [5]–[7]). In principle, dioecy could instead evolve from hermaphroditism via androdioecy, where males first invade a population [5], although the few known instances of androdioecy have probably evolved from dioecy [8]. Irrespective of their evolutionary origin, however, both gynodioecy and androdioecy represent sexual systems in which hermaphrodites are maintained with a single class of unisexual individual. This raises the interesting question of whether the other class of unisexual individual could invade (or re-invade), i.e., whether pure males or pure females could invade a gynodioecious or androdioecious population, respectively. Such a trimorphism has been labeled ‘trioecy’ [9].

Theoretical analysis of the possible evolution and maintenance of trioecy has yielded conflicting expectations about its evolutionary stability. Maurice and Fleming ([10]; this paper is hereafter referred to as MF) showed that trioecy can be maintained under pollen limitation of female seed production because pollen limitation reduces the fitness of females but not self-fertile hermaphrodites, counteracting the seed fertility advantage of females. By contrast, Wolf and Takebayashi ([11]; hereafter referred to as WT) showed that trioecy is not evolutionarily stable for any of the parameter combinations they considered, and the conditions that, for example, enable invasion by females into androdioecious populations are the same as those that result in the displacement of hermaphrodites by females, which yields dioecy (not trioecy). Similar results have also been suggested for potentially trioecious populations of clam shrimps [12]. The key difference between these models involves assumptions about the factors regulating the availability of outcross pollen vs. hermaphrodite selfing. In the MF model, realized hermaphrodite selfing depends on the frequency of females, and not directly on the availability of outcross pollen, which in a trioecious population would also be determined by the frequencies of males and hermaphrodites and their relative pollen fertilities. They further assumed a fixed selfing rate and that non-selfed ovules are lost if they are not outcrossed (i.e., hermaphrodite seed set can be pollen limited). By contrast, in the WT model it is assumed that the availability of outcross pollen is a constant determined by the pollen fertilities of males and hermaphrodites, and that all hermaphrodite ovules not outcrossed are selfed (i.e., hermaphrodite seed set is not pollen-limited).

Although the MF and WT models make different assumptions about how the availability of pollen influences the evolution of sexual systems, both models demonstrate that increased selfing by hermaphrodites reduces the range of conditions that enable the maintenance of unisexual plants, particularly if the magnitude of inbreeding depression is low. The effect of selfing is threefold. First, it promotes the maintenance of hermaphrodites because selfed offspring have no unisexual parents and therefore transmit fewer, if any, female or male-determining genes. Second, selfing by hermaphrodites reduces the number of ovules available for siring by males, reducing the frequency of males in subsequent generations [13]. Third, if selfing occurs in hermaphrodites because of an absence of mating partners and outcross pollen, females in the same setting will tend to show reduced seed set as a result of pollen limitation (e.g., [14]). Thus, low plant densities might disadvantage both males, because of their poorer access to ovules, and females, because of lower pollen availability. The presence of males in a population at high density, by contrast, should be an advantage to females, because males produce more pollen than hermaphrodites [8] and should reduce the degree of pollen limitation experienced by females. Neither model, nor the predictions outlined here, have been tested directly because of the lack of well established examples of trioecy (though see [9], [15]).

**Figure 1:**
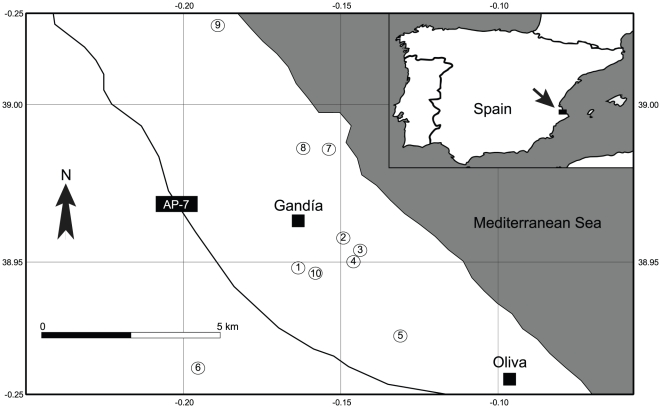
Locations of each of the 10 sites used in this study. The arrow indicates the location of the study region in Spain. Grid lines indicate (decimal) degrees north and east.

Here, we report the existence of trioecious populations of *Mercurialis annua* L. (Euphorbiaceae). *Mercurialis annua* is a wind-pollinated, annual plant found in disturbed and roadside habitats throughout Central and Western Europe as well as around the Mediterranean Basin [16]. The species complex shows striking variation in its sexual systems. Dioecious populations, which are diploid, range from Israel into southern France and northern Spain [17]. In northern Spain, dioecy gives way to monoecy and/or androdioecy in hexaploid populations, which occur through the rest of the Iberian Peninsula and into North Africa [18]. Androdioecious populations comprise hermaphrodites (monoecious individuals) and males with very different inflorescences, i.e., males are not just female-sterile hermaphrodites: staminate flowers on males are held on erect peduncles (or inflorescence stalks), whereas the staminate flowers of monoecious individuals are subsessile in the leaf axils. Inbreeding depression in these hexaploid, androdioecious populations appears to be close to zero [19]. During a survey of sex-ratio variation in androdioecious populations of *M. annua* in eastern Spain, we found substantial numbers of male-sterile hermaphrodites coexisting with fully fertile hermaphrodites and males in several populations near Gandía (Fig. 1).

The male-sterile individuals we observed around Gandía either possessed no stamens (their staminate flowers thus comprising just a single whorl of sepals), or their stamens contained no pollen. We also found apparent homeotic mutants in which the staminate flowers contained sterile pistils in place of stamens. All these phenotypes had previously been identified by Durand [17], but he made no systematic survey of their frequencies. Substantial frequencies of male-sterile hermaphrodites of *M. annua* can be found in male-less populations of the species in various parts of the species range, notably north of Seville in the Sierra Norte, and male sterility is found sporadically in androdioecious populations, with even male individuals sometimes expressing male sterility (J.R. Pannell, unpubl. obs.). In Morocco, in the region surrounding Fes, Durand [17] documented a large number of populations with males, hermaphrodites and females, the latter at appreciable frequencies. These populations are, however, androdioecious, not trioecious: the females represent hermaphrodites with strongly female-biased sex allocation, and the distribution of gender in the populations is bimodal rather than trimodal. The trioecious populations in eastern Spain reported here stand out in terms of the higher frequency of male-sterile individuals they present, their trimodal distribution of gender, and the fact that they were concentrated in several populations over a relatively small geographic region.

Previous work on *M. annua* has confirmed theoretical expectations [20] that males can be maintained with hermaphrodites as long as males are able to sire a sufficient proportion of the ovules produced by the hermaphrodites [13]. Male siring success, and thus the conditions for their maintenance, is enhanced when hermaphrodites self-fertilize fewer of their progeny [13]. In *M. annua*, hermaphrodite selfing is largely determined by the local availability of outcross pollen, which in turn is affected by the density of pollen-producing plants within populations. For example, in experimental mating arrays, an increase in interplant distances of hermaphroditic *M. annua* plants from 22 cm to 33 cm caused the selfing rate to increase from about 0.4 to 0.8 [21]. Because the maintenance of females with males and hermaphrodites should also be influenced by the availability of outcross pollen [MF, WT], we evaluated whether female frequencies in natural populations were associated with the local density of plants. We then considered the conditions that might allow the maintenance of females in populations with males and hermaphrodites, thereby evaluating predictions for the stable maintenance of trioecy. To test these predictions, we first measured the seed production of females relative to hermaphrodites in common gardens set up at different densities and with different male frequencies. We then estimated the frequency of males, females and hermaphrodites in the progeny produced by mating in the common gardens. We predicted that females would be more highly represented among progeny in populations grown at high density and with a high frequency of males.

## Materials and Methods

### Sampling natural populations

We examined variation in female, male, and hermaphrodite frequencies across populations at ten sites (Fig. 1). At each site, two subsites representing the extremes of local plant densities were chosen for surveys of variation in the sex ratio (range of plant densities = 1.4–861 plants/m^2^, average = 203 plants/m^2^). At each of these subsites, sex ratios were evaluated from samples of at least 100 plants (range = 100–374 plants, average = 157 plants). We evaluated densities from plots of sufficient size to exceed the target of 100 plants per subsite. Because plant densities varied across subsites, different sized plots were used at each site, ranging from 60 cm×60 cm at high plant densities, to 8 m×10 m at low plant densities. From this sample of plants, seeds from hermaphrodites and females were harvested separately, yielding collections of seeds from each of 20 subsites and from each of two sexes per subsite.

### Common garden experiment

In May 2008, seeds from all ten source populations were germinated in *Premier Pro-Mix PGX* (Premier Tech Horticulture, Rivière-du-Loup, QC) and grown under greenhouse conditions at Trent University, Peterborough, Ontario. Approximately 200–300 seeds from each sex-by-subsite combination were sown in 10 cm pots. Following germination, seedlings were individually transplanted into 200-cell horticultural trays, with a volume of 11.5 cm^3^/cell. Sex ratios were recorded from these plants as they matured (plants of *M. annua* begin flowering quickly after establishment; flowers are usually produced in the second set of leaf axils arising on the main shoot).

These first-generation plants were transplanted outdoors into 20 raised beds in an open field area at Trent University. Two levels of a density treatment were used to evaluate the effect of plant densities on the maintenance of females. Accordingly, half of these beds were 2.4 m×2.4 m, into which 49 plants were transplanted (i.e., seven rows of seven plants, yielding a final density of 8.5 plants/m^2^), henceforth known as “low-density plots”. The remaining plots were 1.2 m×1.2 m, and received 225 plants (i.e., 15 rows of 15 plants, yielding a final density of 156.25 plants/m^2^), henceforth known as “high-density plots”. The transplanted plants were randomly selected from the progeny of the natural populations grown in the greenhouse, with the following constraint: because the genetic basis of male sterility in this species is not known, we endeavored to standardize the genetic backgrounds of the females, males and hermaphrodites grown in the experiment by randomly selecting female transplants from female mothers and randomly selecting hermaphrodite and male transplants from hermaphrodite mothers. To further evaluate the effect of pollen availability on the maintenance of females, we crossed the density treatment with a male presence/absence treatment. This yielded five replicate plots for each density×male treatment combination. In half of the plots, males were included to yield a sex ratio of 1 hermaphrodite∶1 female∶1 male. For the five high-density plots with males, 75 hermaphrodites, 75 females, and 75 males were grown together, and in the five low-density plots 17 hermaphrodites, 16 females, and 16 males were grown together (an extra hermaphrodite was used in the low-density plots to maintain an even distribution of plants within plots). Males were excluded from the remaining plots, which had sex ratios of 2 hermaphrodites∶1 female (i.e., five low-density plots with 33 hermaphrodites∶16 females, and five high-density plots with 150 hermaphrodites∶75 females). These frequencies were chosen to keep the proportion of females constant among plots, and thereby simplify statistical evaluation of changes in females frequencies in the next generation. These female frequencies also corresponded quite closely with the average frequency of females surveyed in the high-density subsites (see Results). Plants within each plot were evenly spaced in randomized positions keeping the proportions of plants from each source population constant across plots. Following Dorken and Pannell [13], plots were separated from one another by a minimum of 2 m and surrounded by a 1 m high wall of white corrugated PVC plastic to reduce gene flow among plots (it is known from separate experiments that pollen flow between plants decreases significantly when plants are separated by 30 cm or more, thus a difference of 2 m was expected to strongly limit gene flow between plots [21]).

Measurements of the pollen and seed production of the plants from each plot were made after four weeks of growth in the outdoor plots. We measured the allocation to male function by males and hermaphrodites from a random sample of 20 plants from each plot. For trioecious plots, we measured the male allocation of 10 males and 10 hermaphrodites, and in gynodioecious plots we measured the male allocation of 20 hermaphrodites. Following Pannell [22], for each plant, we removed all of the staminate flowers and separately dried and weighed the flowers and vegetative parts. The staminate flowers of males and hermaphrodites are effectively identical, and pollen accounts for 55% of their biomass in both morphs [23], so that staminate floral biomass provides a useful surrogate for male investment. We similarly measured the female allocation of females and hermaphrodites from a random sample of 20 plants from each plot (10 females and 10 hermaphrodites from each trioecious and gynodioecious plot, using the same hermaphrodites for which pollen production had also been measured). For each of these plants, we separately weighed seeds and vegetative parts of the plant that had been air-dried at room temperature in perforated plastic bags. Drying the plants in perforated plastic bags was necessary so that viable seeds could be collected from the plants and this procedure was applied to all plants for which pollen and/or seed production was measured. We defined the pollen production of hermaphrodites and males as the proportion of above-ground biomass allocated to pollen (for hermaphrodites this is denoted as *π*_h_, for males as *π*_m_). Similarly, we defined the seed production of hermaphrodites and females as the proportion of above-ground biomass allocated to seeds (denoted below as *σ*_h_ for hermaphrodites, and as *σ*_f_ for females). Thus seed and pollen production, as defined here, represent the reproductive effort of plants through their female and male functions, respectively. The seeds of the remaining plants from each plot were harvested in bulk.

The following spring, seeds that had been collected from each plot were combined into separate containers and thoroughly mixed. From each plot, we planted 600 seeds into the cells of three 200-cell horticultural trays. Plants were grown under uniform greenhouse conditions at Trent University until they reached sexual maturity. For each plant, we recorded its sex following five weeks of growth in the greenhouse. Germination rates averaged approximately 50% and we estimated the sex ratios from a total of 6076 progeny for an average of 304 plants per plot (range = 243–337).

### Data analyses

To evaluate differences in female, male and hermaphrodite frequencies among the natural trioecious populations based on the effect of density, we used a generalized linear mixed model (GLMM), with population included as a random grouping variable. The proportion of female plants was the dependent variable, and the local density of plants at each subsite (coded as a categorical variable with values “high” or “low”) was the independent variable. The model was fitted by specifying binomial errors and a logit link function using the lmer function in the lme4 library [24] in R [25]. To determine differences in allocation to seed production between hermaphrodites (*σ*_h_) and females (*σ*_f_), we used a linear mixed-effects model using the lme function in the nlme library [26]. Density, male frequency and sex (and all possible interactions) were included as fixed effects, and plot was considered a random effect. We similarly used a linear mixed-effects model to evaluate the effect of density and plant sex (and their interaction) on the pollen production of hermaphrodites (*π*_h_) and males (*π*_m_). Plot was considered a random effect and the model was again calculated using the lme function. The effect of plant densities and male frequencies on the frequency of females, males, and hermaphrodites in the next generation was evaluated using generalized linear models (using the glm function in the MASS library [27]) with density and male presence/absence and their interaction considered fixed effects and using quasibinomial errors to account for overdispersion [28]. Note that tests of changes in male frequencies among the progeny only involved those plots with males present, and so only the density treatment was included as a fixed effect. To evaluate differences in the frequency of females among the parents vs. the progeny of each plot we used replicated goodness-of-fit tests [29], using each plot as the unit of replication and each treatment combination to group each set of tests.

## Results

### Natural populations

**Figure 2:**
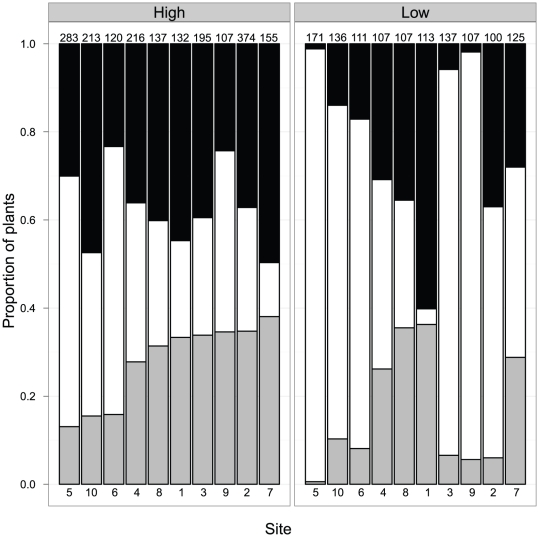
Variation in the frequency of females (grey bar segments), hermaphrodites (white bar segments), and males (black bar segments) at each high density (left panel) and low density (right panel) subsite. Subsites in each panel are ordered by the local frequency of females in the high-density subsites. Numbers at the top of each bar indicate the total sample size per subsite. Numbers at the bottom of each bar correspond with the site numbers in Fig. 1.

In natural populations, female frequencies were, on average, 75% higher in high-density subsites than they were in low-density subsites (average proportion of females in high-density subsites ±1 S.E. was 0.28±0.03 plants/m^2^ vs. 0.16±0.04 in low-density subsites; GLMM Wald's *Z* = −6.28, *P*<0.001; Fig. 2). Male frequencies were also substantially higher in high- compared to low-density subsites (average proportion ±1 S.E. of males in high-density subsites: 0.37±0.03, low-density subsites: 0.23±0.06; GLMM Wald's *Z* = −7.08, *P*<0.001; Fig. 2). There was a corresponding decrease in the proportion of hermaphrodites in high-density subsites (GLMM Wald's *Z* = 8.51, *P*<0.001; average proportion of hermaphrodites in high-density subsites: 0.35±0.05; low-density subsites: 0.60±0.10).

### Experimental plots

**Figure 3:**
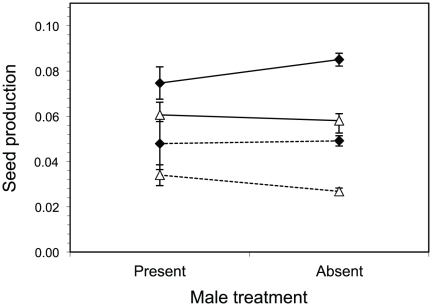
Differences in the proportion of above-ground biomass allocated to seeds between females (*σ_f_* - diamonds) and hermaphrodites (*σ_h_* - triangles) in plots with and without males. Means (± S.E.) of plants grown under high densities are joined with a solid line, and with a dashed line for plants grown under low densities.

In the experimental plots, *σ_f_* was 42% greater than *σ_h_* (average *σ_f_*±1 S.E. across density levels was 0.061±0.002 vs. *σ_h_* = 0.043±0.002; Table 1 and Fig. 3); i.e., female reproductive effort was greater than that for the female component of hermaphroditic reproductive effort. Density also influenced patterns of reproductive effort towards seed production by females and hermaphrodites. In high-density plots, female reproductive effort by females and hermaphrodites was 74% more than in low-density plots (high density plots: average *σ* for females and hermaphrodites ±1 S.E. was 0.069±0.002 vs. low density *σ* = 0.040±0.001). There was a significant interaction between sex and male frequency on patterns of seed production. For females, reproductive effort was 9% higher in the absence of males (males absent: *σ_f_* = 0.067±0.008 S.E. vs. males present: *σ_f_* = 0.061±0.006 S.E.), whereas its female component in hermaphrodites was 12% lower in the absence of males than it was in their presence (males absent: *σ_h_* = 0.042±0006 S.E. vs. males present: *σ_h_* = 0.047±0.002 S.E.).

**Table 1:** Linear mixed-effects models used to evaluate patterns of allocation to seed production by females (*σ*_f_) and hermaphrodites (*σ*_h_) and the allocation to pollen production by males (*π*_m_) and hermaphrodites (*π*_h_).

Effect	Seed production	Pollen production
Density	*F*_1,16_ = 38.4***	*F*_1,18_ = 24.7**
Male Frequency	*F*_1,16_ = 0.02	N/A
Sex	*F*_1,445_ = 89.1***	*F*_1,310_ = 1632.5***
Density×Male Freq.	*F*_1,16_ = 0.3	N/A
Density×Sex	*F*_1,445_ = 0.1	*F*_1,310_ = 1.2
Male Freq.×Sex	*F*_1,445_ = 6.3*	N/A
Density×Male Freq.×Sex	*F*_1,445_ = 0.3	N/A

* *P*<0.05;

** *P*<0.001;

*** *P*<0.0001.

**Figure 4:**
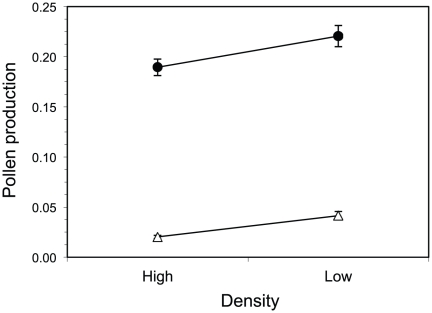
Differences in the proportion of above-ground biomass allocated to pollen between males (*π_m_* - circles) and hermaphrodites (*π_h_* - triangles) in high and low density treatments. Error bars represent one standard error.

Male reproductive effort by both males and hermaphrodites was influenced by density and sex (i.e., male vs. hermaphrodite; Table 1; note that the effect of male treatment could not be evaluated for pollen allocation because males were present only in one of the treatment combinations). Across treatments, reproductive effort by males was, on average, almost 6× higher than the male component of hermaphrodites (*π_m_* = 0.205±0.005 S.E. vs. *π_h_* = 0.035±0.002 S.E.; Fig. 4). Male reproductive effort was 24% greater in low-density plots than in high-density plots (average *π* for males and hermaphrodites in low-density plots = 0.103±0.006 S.E.; in high-density plots = 0.078±0.021 S.E.). There was no interaction between plot density and sex for pollen production (Fig. 4).

In the second generation, a generalized linear model did not reveal any overall effect of plant densities or male frequencies on the frequency of females (Density: *t* = 0.7, *P*>0.50; Male frequency: *t* = 0.1, *P*>0.85; Density×Male frequency: *t* = −0.5, *P*>0.50). Considering only the plots with males, plant densities had no effect on the frequency of males in the second generation (Density: *t* = −0.54, *P*>0.50). Plant densities also had no effect on the frequency of hermaphrodites in the second generation (Density: *t* = 0.72, *P*>0.25; Male frequency: *t* = 7.92, *P*<0.001, Density×Male frequency: *t* = −1.04, *P*>0.25; n.b., the significant effect of male frequencies reflects the higher overall frequency of hermaphrodites in the parental generation in plots without males).

**Figure 5:**
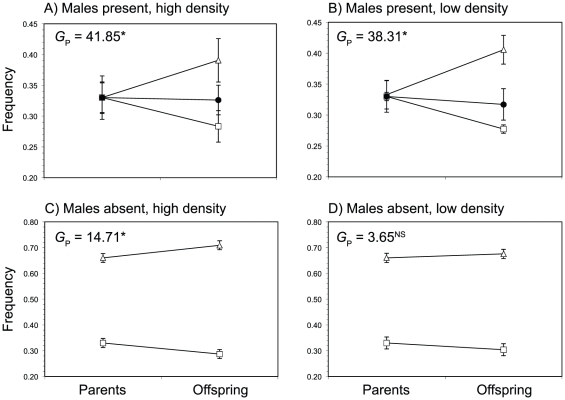
Parental and progeny sex ratios of females (squares), hermaphrodites (triangles), and males (circles) in high density treatments with males (A), without males (C), and low density treatments with males (B) and without males (D) (a small proportion of males were found in the low density with no males treatment, but this is not depicted). Results of the replicated goodness of fit tests from Table 2 (pooled for each density by male treatment combination) are shown in each panel (* *P*<0.0001; ^NS^ *P*>0.05).

The replicated goodness-of-fit tests revealed substantial heterogeneity in the frequency of the three sex phenotypes in the next generation (Table 2; Fig. 5). However, in spite of this heterogeneity, these tests still indicated significant overall changes in sex phenotype frequencies across generations (see the values for *G*_P_ in Table 2). In general, significant values of *G*_P_ were associated with increases in the frequency of hermaphrodites. Indeed, for high-density plots grown without males, the significant value for *G*_P_ indicates that the frequencies of females and hermaphrodites diverged from one generation to the next (with a decline in female frequencies). By contrast, and contrary to our expectations, there was no evidence for an overall increase in hermaphrodite frequencies for low-density plots from which males had been excluded. However, there was significant heterogeneity among these plots, and, for at least one plot, a significant excess of hermaphrodites was detected. Note that low frequencies of males were detected among the progeny of plants grown without males, particularly for the low-density plots. In *M. annua* males only occur among the progeny of plants involving male sires [30], therefore this observation indicates a small degree of pollen dispersal among plots.

**Table 2:** Frequency of males, hermaphrodites, and females among the progeny of plants grown under high- vs. low-density conditions and in the presence vs. absence of males.

Plot	Density	Males	*p*_h_	*o*_h_	Male	Herm	Female	*n*	*G*
6	Low	Absent		0.60	0.01	0.62	0.37	337	2.87
9	Low	Absent		0.49	0.01	0.68	0.31	332	0.56
11	Low	Absent		0.49	0.02	0.69	0.29	308	2.14
14	Low	Absent		0.48	0.02	0.66	0.32	304	0.17
16	Low	Absent		0.70	0.04	0.73	0.23	326	12.57**
1	Low	Present	0.20	0.34	0.37	0.36	0.27	304	5.05
3	Low	Present	0.21	0.73	0.24	0.46	0.30	311	21.56***
8	Low	Present	0.14	0.71	0.33	0.38	0.29	320	4.57
17	Low	Present	0.22	0.91	0.27	0.47	0.26	316	23.09***
19	Low	Present	0.17	0.88	0.37	0.36	0.27	243	4.57
2	High	Absent		0.75	0.01	0.71	0.28	320	3.30
5	High	Absent		0.83	0.00	0.70	0.29	297	2.14
7	High	Absent		0.61	0.00	0.77	0.22	309	17.43***
10	High	Absent		0.73	0.00	0.69	0.31	302	0.98
20	High	Absent		0.60	0.00	0.67	0.33	285	0.02
4	High	Present	0.13	0.73	0.27	0.42	0.31	298	9.43*
12	High	Present	0.11	1.03	0.36	0.29	0.35	297	2.23
13	High	Present	0.08	0.79	0.33	0.36	0.32	304	0.68
15	High	Present	0.13	0.75	0.27	0.51	0.22	291	38.11***
18	High	Present	0.08	0.87	0.40	0.38	0.22	272	24.16**

Averages								*G*_H_	*G*_P_
Low	Absent			0.55	0.02	0.68	0.30	14.65*	3.65
Low	Present		0.19	0.71	0.32	0.41	0.27	20.53**	38.31***
High	Absent			0.70	0.00	0.71	0.29	9.16	14.71***
High	Present		0.11	0.83	0.33	0.39	0.28	24.16***	41.85***

Shown are the relative pollen production of hermaphrodites versus males (*p*_h_, where *p_h_* = π_h_/π_m_), the relative seed production of hermaphrodites versus females (*o_h_*, where *o_h_* = σ_h_/σ_f_), the frequency of each sex in the next generation, and the sample size. *G* values are given for tests of independence of sex frequencies from the parental versus progeny generation for each plot. *G*_H_ refers to tests of heterogeneity between plots in the same treatment. *G*_P_ combines data from all plots, testing for overall changes in the frequency of the three sex phenotypes.

* *P*<0.05;

** *P*<0.01;

*** *P*<0.001.

A small proportion of neuter males (both male- and female-sterile) were detected among the progeny grown in the greenhouse, on average comprising between 0% and 3.0% of plants in each density×male frequency treatment group. However, these differences in the frequency of neuter males were not statistically significant (data not shown). In Table 2, and the associated GLM and replicated goodness of fit tests, neuter males were included as males.

## Discussion

### No effect of density on male or female frequencies

We observed highly variable female frequencies among natural populations of trioecious *M. annua*. Some of this variation appeared to be governed by patterns of local plant densities. Specifically, high female frequencies were associated with high plant densities, coincident with the conditions that also favour the maintenance of males [13]. By contrast, in our experiment we detected no effect of an 18-fold difference in plant densities on evolutionary trajectories of female frequencies from one generation to the next. Previous work on androdioecious *M. annua* pointed to a clear negative relationship between the selfing rate of hermaphrodites and plant density, and we expect a similar density-dependence for hermaphrodite mating in our common gardens here. The previous study found that *M. annua* hermaphrodites separated by more than 30 cm from another pollen-producing individual self-fertilized almost all of their ovules [21], suggesting that outcross pollen becomes substantially diluted at these inter-plant distances. Plants in our low-density plots were separated by slightly more than 30 cm, so that hermaphrodites were probably selfing at relatively high rates. Under these conditions, hermaphrodites ought to have increased in frequency over females and males. However, in our experiment, hermaphrodite frequencies increased in all plot types, while female frequencies declined. This finding, which is discussed in more detail below, contrasts with a previous study on *M. annua* [13], in which hermaphrodite frequencies increased in experimental androdioeious populations of *M. annua* grown at low, but not high densities.

Also in stark contrast to the results of Dorken and Pannell [13], who found that male frequencies declined when grown under low densities, probably because of selfing by hermaphrodites, we found no effect of density on the proportion of males in the next generation. The main difference between the two experiments was the presence of high frequencies of females in all of our plots here. The presence of females was likely to have buffered the effect of density-dependent selfing by hermaphrodites on male siring success by providing a large number of ovules available for outcrossing regardless of plant densities. Males will have sired a large fraction of these because they produced so much more pollen than hermaphrodites [13], [22].

Densities could also have influenced the relative seed production of females vs. hermaphrodites if hermaphrodites express phenotypic plasticity of sex allocation in response to plant density. Phenotypic plasticity of hermaphrodite sex allocation is common among flowering plants and has been shown to regulate the seed production of females vs. hermaphrodites in gynodioecious species [31]–[33]. Previous experiments on *M. annua* have shown that hermaphrodites increase their relative allocation to female function when grown under higher densities [13]. Therefore, if females maintain constant allocation to seed production, and hermaphrodites alter their allocation across a gradient of plant densities, we might have expected this to have altered the seed fertilities of females vs. hermaphrodites in our experiment. We did detect the expected decrease in the seed production of hermaphrodites in low-density plots, but the difference in the seed allocation of plants under low- vs. high densities was similar for both females and hermaphrodites, because females also reduced their allocation to seed production under these conditions. There is thus no evidence that sex-differential plasticity in *M. annua* should contribute to the regulation of female frequencies in trioecious *M. annua*.

Hermaphrodites, but not females, increased their seed production when grown with males. Because *M. annua* hermaphrodites are self compatible and not pollen-limited when grown in the absence of males [34], this observation is not likely to have been the result of enhanced seed production via higher levels of pollen deposition. Instead, these results appear to be similar to those from *Begonia gracilis*, a monoecious plant that increases its allocation to female function in response to higher levels of pollen deposition [35]. For the hermaphrodites in our experiment, such responses have the potential to be adaptive, because they enable facultative adjustment of sex allocations in response to the operational sex ratio during mating [36]. By contrast, females necessarily allocate all of their reproductive resources to seed production, and therefore lack the capacity for facultative adjustment of sex allocations. The fact that hermaphrodites reduced their female reproductive effort in the absence of males could be attributable to the effects of increased selfing and inbreeding depression. However, this seems unlikely, given that Iberian populations of *M. annua* express almost no inbreeding depression [19], [37].

### Hermaphrodite selfing and the maintenance of trioecy

The ability of hermaphrodites to self-fertilize their ovules, particularly in the absence of other mates, is a key factor regulating the evolution of plant sexual systems [5], [6], [20]. This ability is central to both the MF and WT models, but in spite of this commonality, the two models yield divergent expectations for the evolutionary stability of trioecy. Attempting to apply the MF model to trioecious *M. annua* is probably not appropriate because, in that model, selfing is assumed to be regulated by the frequency of females. However, outcrossing in wind-pollinated *M. annua* occurs via a process akin to scramble competition between self vs. outcross pollen grains. Self-pollen has the advantage of closer proximity to female flowers on the same plant, but in high-density populations, this advantage is swamped by the abundance of pollen produced by neighbouring plants [21]. Thus, selfing in *M. annua* is regulated by the frequency and density of pollen-producing plants (i.e., the males and hermaphrodites), not the frequency of females. Because the pollen production of males and hermaphrodites should usually differ substantially, using the frequency of females as a proxy for the degree to which plants might be pollen-limited is most appropriate when there are only two sex phenotypes (i.e., dioecious or gynodioecious populations, for which the expression used by MF was initially developed [38]).

The assumptions made by WT regarding the availability of outcross pollen more closely match the biology of trioecious *M. annua* than those of MF. In their model, WT assume that outcrossing opportunities are constant, or are a function of the male and hermaphrodite frequencies (weighted by the pollen production of each sex), and that hermaphrodites are not pollen limited. Moreover, they have shown that under nuclear inheritance of sex expression, females can only invade androdioecious populations under high rates of outcrossing. The threshold value of the outcrossing rate regulating female invasion depends on the seed and pollen production of hermaphrodites relative to females and males, respectively, the selfing rate, and the viability of selfed vs. outcrossed seeds as follows (their equation 11):
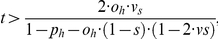
where *t* is the outcrossing rate, *s* is the (prior) selfing rate, *o_h_* is the ovule production of hermaphrodites relative to females (i.e., *o_h_* = σ_h_/σ_f_), *p_h_* is the pollen production of hermaphrodites relative to males (*p_h_* = π_h_/π_m_) and *v_s_* is the viability of selfed relative to outcrossed seeds. Using data from this and previous experiments, we can infer whether this threshold value of *t* is ever likely to be exceeded in natural populations of *M. annua*. Our results provide values for the relative seed and pollen fertilities of hermaphrodites relative to unisexuals. Previous experiments involving *M. annua* have shown that inbreeding depression is close to 0 in Iberian populations (i.e., *v_s_* is close to 1.0; [19], [37]). Finally, the influence of plant densities on selfing rates (*s*) has been measured across a range of plant densities similar to those used in this study (i.e., we can infer that *s* was probably around 0.8 and 0.2, respectively, under low densities and high densities in our experiment here [21]). For this combination of parameter values, and under the experimental conditions we applied, there would appear to be no biologically realistic values of the outcrossing rate that would lead to the maintenance of females; even with *t* = 1.0, females cannot be maintained and should always decrease in frequency, as was observed in our arrays.

The above formulation assumes that male sterility is caused by a mutation segregating in the nuclear genome and thus transmitted through both seeds and pollen. However, male sterility in many gynodioecious species is due to mutations transmitted only through ovules and seeds (usually in the mitochondrial genome), and under these conditions females can be maintained under less stringent conditions [39], [40]. In particular, under male sterility transmitted by cytoplasmic genes, females can typically be maintained with hermaphrodites, even in the absence of selfing and inbreeding depression, if *o_h_*<1.0 (as opposed to *o_h_*<0.5 when male sterility is inherited though nuclear genes). These conditions appear to be met in our experiment.

We do not yet know how male sterility in *M. annua* is inherited, but it is possible that cytoplasmic factors play a role. Even so, the fact that female frequencies declined in our experiment indicates either that we were unable to emulate conditions that might maintain them in the field, or that they should eventually be lost in the field, too. In the latter case, we would need to invoke drift to account for their existence at reasonable frequencies in natural populations. Given that *M. annua* is a ruderal species subject to large fluctuations in population size ([41]; M.E. Dorken, R.P. Freckleton, and J.R. Pannell, unpublished data), a scenario invoking drift to explain high female frequencies in some populations seems reasonable. Even their occurrence at an elevated frequency regionally, as observed around Gandía, might be explained by drift if the regional genetic effective size, *N*_e_, of *M. annua* is sufficiently small. A process of frequent population turnover in a metapopulation, as has been invoked to explain sex-ratio variation [22], [41] and patterns of neutral genetic diversity in *M. annua* [42], is one scenario known to drastically reduce *N*_e_ regionally [43]–[45].
